# Association between successful aging transitions and depressive symptoms among older Korean adults: findings from the Korean longitudinal study of aging (2006–2018)

**DOI:** 10.1186/s12877-021-02250-6

**Published:** 2021-06-09

**Authors:** Fatima Nari, Bich Na Jang, Selin Kim, Wonjeong Jeong, Sung-In Jang, Eun-Cheol Park

**Affiliations:** 1 Department of Public Health, Graduate School, Yonsei University, Seoul, Republic of Korea; 2 Institute of Health Services Research, Yonsei University, Seoul, Republic of Korea; 3 Department of Preventive Medicine, Yonsei University College of Medicine, 50 Yonsei-to, Seodaemun-gu, 03722 Seoul, Republic of Korea

**Keywords:** Successful aging, older adults, longitudinal analysis, depressive symptoms, KLoSA

## Abstract

**Background:**

The importance of SA (Successful aging) has been emphasized in recent years, with focus shifting towards attaining healthier aging rather than longevity. However, the influence of SA and its changes on mental health such as depression remains a relatively unexplored area in gerontology. Therefore, we investigated the longitudinal association between changes in SA and depressive symptoms in Korean older adults.

**Methods:**

This study comprised a longitudinal sample of older adults aged ≥ 45 years, drawn from the Korean Longitudinal Study of Aging (2006–2018). Changes in SA status was determined using the Rowe and Kahn model over two consecutive years. Using an adjusted generalized estimating equation model, we examined the association between changes in successful aging status, namely SA and NSA (Non-successful aging), and depressive symptoms.

**Results:**

Compared to the SA→SA group, depressive symptom risk in the NSA→NSA and SA→NSA groups were higher in men [(OR, 1.16; 95 % CI, 1.13–1.18), (OR, 1.11; 95 % CI, 1.08–1.13), respectively] and in women [(OR, 1.15; 95 % CI, 1.13–1.18), (OR, 1.11; 95 % CI, 1.09–1.14), respectively]. Subgroup analysis of the dimensions of successful aging revealed that low or worsening criteria of successful aging status in men and women were associated with depressive symptoms.

**Conclusions:**

Korean older adults who continuously failed to attain or maintain successful aging status had the highest risk of depressive symptoms. These results could further assist in establishing policies and interventions that promote successful aging and subsequently protect the mental health of the Korean older adult population.

## Background

As the average life expectancy worldwide increases, and we progress towards a more aged society, the world has shifted its focus to tackling the burden that comes along with an aging demographic as well as describing and defining processes leading to healthier, more successful aging. The model of SA is multidimensional and refers to aging healthily despite aging-related challenges [1].

There is no consensus on the exact characterization of SA. Nevertheless, a plethora of studies have adopted the Rowe and Kahn model of successful aging, often with a few minor alterations to the model [2]. The Rowe and Kahn model defines successful aging as having: (1) no major chronic diseases or disability, (2) high physical and cognitive functioning, and (3) an active social life [3].

Previous studies have shown that successful aging is not a final state, but an ever-changing continuum of adaptation and development in the physical and psychosocial context [4–6]. In addition, Stowe et al. discussed the wide and prolific use of the Rowe and Kahn model, stating that its use still has great implications in current gerontological research, albeit requiring a few modifications [7]. Additionally, the authors named the static view of the Rowe and Kahn model as one of its weak points, emphasizing that SA should be viewed as a lifelong and dynamic process [7]. Although many existing studies considered SA as fixed end-point criteria [8, 9], recent research has begun to shift focus towards the dynamic aspect of SA, suggesting that SA undergoes various changes and trajectories over time. A study by Bosnes et al. highlighted the importance of aging and SA, and monitored SA temporally, looking at components of the Rowe and Kahn model, individually and combined [10].

Due to the large proportion of older adults in South Korea, research regarding successful and healthy aging has been increasing over the years. Successful aging in Korea, as defined by Rowe and Kahn’s model, was found to be linked to decreased risk of mortality [11] and better mental health outcomes such as improved life satisfaction [12]. Another recent study by Nakagawa et al. compared the prevalence of successful aging between Japan, Korea, and China using the Rowe and Kahn model [13]. The estimated prevalence of successful aging in Korea was found to be 25.5 % [13].

Increasing evidence shows that mental illnesses and aging trajectories share overlapping pathological pathways, and faster rate of biological aging or “accelerated aging” often coexists with psychiatric disorders such as depression [14]. Depression in older age is of great public health significance and is linked to increased suicide, mortality, and health deterioration [15]. The prevalence of depressive disorders in the Korean older population was reported to be higher than in their western counterparts [15], and the prevalence was estimated to be 35.4 % in those older than 80 years [15, 16].

Prior research looked at depression as a predictor of SA [17], as well as the cross-sectional relationship between individual subcomponents of SA and depression [18]. Nevertheless, considering the sizable prevalence of SA and depressive symptoms in Korea, it is necessary to explore how the overall SA framework influences depressive symptoms over time compared to non-successful aging to address gaps in knowledge. We hypothesized that SA classified by meeting Rowe and Kahn’s criteria may change over time, and older adults not meeting SA criteria may experience greater risk of depressive symptomology. Thus, the primary aim of this study was to examine the longitudinal association between changes in successful aging status and depressive symptoms in the Korean older population.

## Methods

This study derived data from the 1st to the 7th wave of the Korean Longitudinal Study of Aging (KLoSA) from 2006 to 2018. The KLoSA is an ongoing, large-scale longitudinal study aimed at tackling the emerging health and social burden associated with rapid population aging. Nationally representative data were collected and ensured by using multistage stratified probability sampling on Korean residents aged ≥ 45 years. In 2006, 10,254 respondents were interviewed in the original baseline sample. More detailed information regarding the KLoSA survey can be found on the panel website (https://survey.keis.or.kr/eng/klosa/klosa01.jsp).

**Fig. 1:**
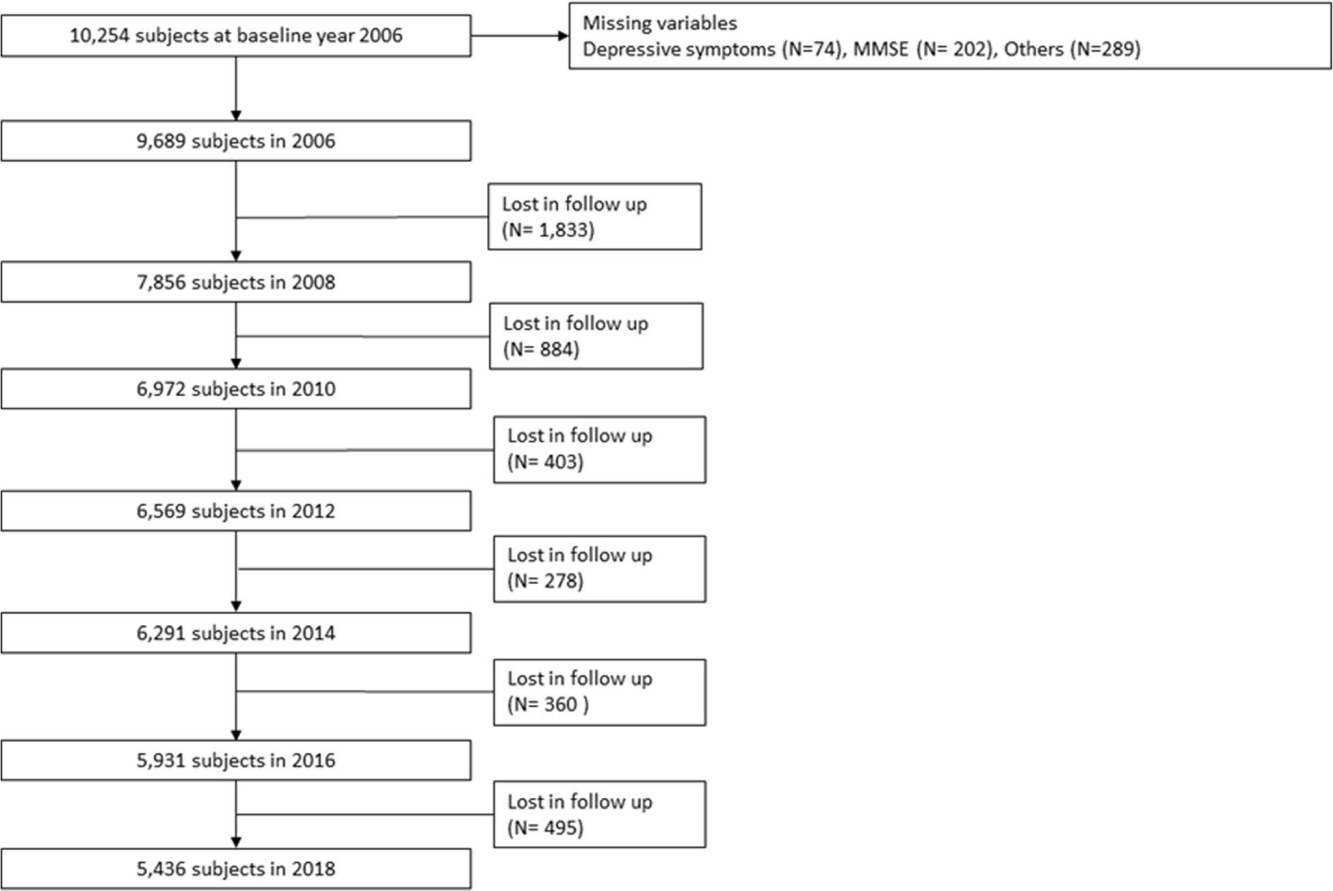
Flowchart of the study participants from 2006–2018

After excluding those who failed to follow up as well as those with missing or incomplete data, 9,689 participants were included in 2006; 7,856 in 2008; 6,972 in 2010; 6,569 in 2012; 6,291 in 2014; 5,931 in 2016; and 5,436 in 2018. The flowchart of participants and the selection process are shown in detail in Fig. 1.

The main outcome measure in our study was depressive symptoms. We followed the criteria set for depression by the Center for Epidemiologic Studies [19]. The KLoSA uses CES-D10, which is the validated, concise version that consists of 10 items listed in the original 20-item version [20]. The total sum of the depression score was 10 points, with higher scores indicating higher depressive symptoms, and we used the recommended cut-off score of 3 points to indicate depressive symptomatology at baseline [21].

Our study’s key independent variable was the change in successful aging status. We utilized the Rowe and Kahn Model to define the criteria for successful aging, “absence of disease and disease-related disabilities,” “maintenance of high mental and physical function,” and “continued engagement with life,” while excluding chronic diseases. The rationale behind this decision was that we did not find it feasible to investigate the change in the absence of chronic diseases. We followed the characterization and operationalization of successful aging implemented by prior studies in Korea [11], albeit with a few alterations. For the “absence of disability” domain, activities of daily living (ADL) and instrumental activities of daily living (IADL) were used. The respondents were classified as being free from disabilities if they had no difficulties in performing ADL, and if they had more than one limitation in the IADL. For the “maintenance of high mental and physical function” domain, cognitive function as representative for high mental functioning, and handgrip strength as an indicator for high physical function were used. The Korean version of the Mini Mental State Examination (MMSE-K) was used to assess if the subjects’ cognitive functions were normal; a score lower than 24 indicated impaired cognitive function [11]. Handgrip strength was dichotomized based on the median value of gender-specific handgrip strength [11, 22]. The “continued engagement with life” domain was determined by asking respondents if they participated in any social activities, and they were considered socially engaged if they partook in at least one social activity, including religious activities, volunteering activities, leisure/sports activities, alumni associations, political parties, and nongovernmental organizations [11]. The operationalization of our main independent variable is further explained in Table 1. The subjects were classified as “SA” if they satisfied all five indicators of the three domains [11, 23], otherwise they were categorized as “NSA”. Changes in SA status were investigated by measuring whether SA was present in the prior year and if the conditions for SA were still met in the succeeding year following a 2-year interval. On this basis, we recognized four different SA profiles and categorized respondents into the following groups: NSA→NSA, SA→NSA, NSA→SA, and SA→SA.

**Table 1:** Definition of Successful Aging according to the Korean Longitudinal Study of Ageing

Domain	Variables	Successful Aging (SA)	Non-Successful Aging (NSA)
Absence of disability	ADL	0	≥ 1
	IADL	0–1	≥ 2
Maintenance of high mental and physical function	MMSE-K	≥ 24	< 24
	HG	Gender-specific median HG value (Men: ≥28.75, Women: ≥17)	Below gender-specific median HG value
Continued engagement with life	Social activities	Participation in one or more activity	0

Abbreviation: *ADL* Activities of Daily Living; *IADL* Instrumental Activities of Daily Living; *MMSE-K* Korean form of the Mini Mental State Examination; *HG* Handgrip Strength

Covariates included in our analysis were gender; age (45–54, 55–64, and ≥ 65 years); education level (Middle school degree or lower, High School degree, and University degree or higher); income level per month calculated per wave and divided into quartiles (low, middle low, middle high, and high); marital status (Married and Unmarried); economic activity (Active and Inactive); region (Urban and Rural); physical activity calculated as the total time spent partaking in regular exercise (minutes/week), and if they exercised for ≥ 150 min/week then it was coded as ‘Yes’, otherwise it was coded as ‘No’; drinking (Current, Past, and Never); smoking (Current, Past, and Never); BMI (Overweight ≥ 25 kg/m^2^, Normal 18.6–24.9 kg/m^2^, and Underweight < 18.6 kg/m^2^); and number of chronic diseases (including hypertension, diabetes, cancer, lung disease, heart disease, and cerebrovascular disease).

The baseline differences between characteristics of the respondents by gender were determined using the χ2 test. Considering the longitudinal nature of our data, a generalized estimating equation (GEE) model was employed for a repeated measure analysis, and a lag function was applied to determine whether SA was present or absent in the previous and following years. In total, seven waves (2006–2018) were used for the analysis, and repeat measurements were carried out for each individual for up to six times. We investigated the risk of depressive symptoms following a successful aging transition over an interval of two consecutive years. The GEE model was adjusted for confounding effects, using the following covariates: age, educational level, income, marital status, economic activity, region, physical activity, drinking, smoking, and number of chronic diseases. Furthermore, two subgroup analyses were carried out as well to further understand the relationship between SA changes and depressive symptoms; one looked at the combined effects of SA changes and other covariates on depressive symptoms. The other subgroup analysis was conducted to investigate the association of changes of individual components of SA and depressive symptoms. Statistical significance was set at p < 0.05. All statistical analyses were conducted using SAS software (version 9.4; SAS Inc., Cary, NC, USA).

## Results

The respondents’ general characteristics at baseline (2006–2008) are shown in Table 2. In total, 7,856 people were included in the baseline year (3,463 men and 4,393 women). The total prevalence of depression in the participants was 47.5 % for men and 60.1 % for women. Men and women in the SA→SA group comprised 35.2 and 32.9 % of the total study population, respectively.

**Table 2:** General characteristics of study population at baseline (2006→2008) (*N*=7,856)

Variables	Total		Men					Total		Women				
			Depressive symptoms (CES-D10 ≥3)				***P***-value			Depressive symptoms (CES-D10 ≥3)				***P***-value
			Yes		No					Yes		No		
	N	(%)	N	(%)	N	(%)		N	(%)	N	(%)	N	(%)	
Total	3,463	(100.0)	1,645	(47.5)	1,818	(52.5)		4,393	(100.0)	2,639	(60.1)	1,754	(39.9)	
**Change in successful aging**							<.0001							<.0001
NSA→ NSA	1,273	(36.8)	792	(62.2)	481	(37.8)		1,793	(40.8)	1,326	(74.0)	467	(26.0)	
SA→NSA	501	(14.5)	279	(55.7)	222	(44.3)		562	(12.8)	358	(63.7)	204	(36.3)	
NSA→SA	469	(13.5)	182	(38.8)	287	(61.2)		592	(13.5)	314	(53.0)	278	(47.0)	
SA→SA	1,220	(35.2)	392	(32.1)	828	(67.9)		1,446	(32.9)	641	(44.3)	805	(55.7)	
**Age**							<.0001							<.0001
45-54	911	(26.3)	345	(37.9)	566	(62.1)		1,225	(27.9)	559	(45.6)	666	(54.4)	
55-64	1,045	(30.2)	432	(41.3)	613	(58.7)		1,201	(27.3)	650	(54.1)	551	(45.9)	
≥ 65	1,507	(43.5)	868	(57.6)	639	(42.4)		1,967	(44.8)	1,430	(72.7)	537	(27.3)	
**Education level**							<.0001							<.0001
Lower than middle school	1,076	(31.1)	644	(59.9)	432	(40.1)		2,490	(56.7)	1,726	(69.3)	764	(30.7)	
Middle school graduate	596	(17.2)	292	(49.0)	304	(51.0)		724	(16.5)	391	(54.0)	333	(46.0)	
High School graduate	1,197	(34.6)	498	(41.6)	699	(58.4)		981	(22.3)	451	(46.0)	530	(54.0)	
University graduate	594	(17.2)	211	(35.5)	383	(64.5)		198	(4.5)	71	(35.9)	127	(64.1)	
**Income level**							<.0001							<.0001
Low	1,148	(33.2)	674	(58.7)	474	(41.3)		1,762	(40.1)	1,253	(71.1)	509	(28.9)	
Middle Low	835	(24.1)	404	(48.4)	431	(51.6)		964	(21.9)	569	(59.0)	395	(41.0)	
Middle High	812	(23.4)	325	(40.0)	487	(60.0)		934	(21.3)	479	(51.3)	455	(48.7)	
High	668	(19.3)	242	(36.2)	426	(63.8)		733	(16.7)	338	(46.1)	395	(53.9)	
**Marital status**							<.0001							<.0001
Married	3,171	(91.6)	1,448	(45.7)	1,723	(54.3)		3,031	(69.0)	1,659	(54.7)	1,372	(45.3)	
Unmarried	292	(8.4)	197	(67.5)	95	(32.5)		1,362	(31.0)	980	(72.0)	382	(28.0)	
**Economic activity**							<.0001							<.0001
Active	2,053	(59.3)	808	(39.4)	1,245	(60.6)		1,321	(30.1)	675	(51.1)	646	(48.9)	
Inactive	1,410	(40.7)	837	(59.4)	573	(40.6)		3,072	(69.9)	1,964	(63.9)	1,108	(36.1)	
**Region**							<.0001							<.0001
Urban	2,610	(75.4)	1,152	(44.1)	1,458	(55.9)		3,328	(75.8)	1,914	(57.5)	1,414	(42.5)	
Rural	853	(24.6)	493	(57.8)	360	(42.2)		1,065	(24.2)	725	(68.1)	340	(31.9)	
**Physical activity**							<.0001							<.0001
Yes	1,098	(31.7)	444	(40.4)	654	(59.6)		1,167	(26.6)	567	(48.6)	600	(51.4)	
No	2,365	(68.3)	1,201	(50.8)	1,164	(49.2)		3,226	(73.4)	2,072	(64.2)	1,154	(35.8)	
**Drinking**							<.0001							<.0001
Current	2,105	(60.8)	902	(42.9)	1,203	(57.1)		828	(18.8)	440	(53.1)	388	(46.9)	
Past	563	(16.3)	339	(60.2)	224	(39.8)		184	(4.2)	122	(66.3)	62	(33.7)	
Never	795	(23.0)	404	(50.8)	391	(49.2)		3,381	(77.0)	2,077	(61.4)	1,304	(38.6)	
**Smoking**							0.5092							<.0001
Current	1,315	(38.0)	621	(47.2)	694	(52.8)		137	(3.1)	104	(75.9)	33	(24.1)	
Past	918	(26.5)	460	(50.1)	458	(49.9)		43	(1.0)	29	(67.4)	14	(32.6)	
Never	1,230	(35.5)	564	(45.9)	666	(54.1)		4,213	(95.9)	2,506	(59.5)	1,707	(40.5)	
**BMI**							<.0001							0.1892
Overweight	650	(18.8)	257	(39.5)	393	(60.5)		1,051	(23.9)	626	(59.6)	425	(40.4)	
Normal	2,695	(77.8)	1,309	(48.6)	1,386	(51.4)		3,168	(72.1)	1,893	(59.8)	1,275	(40.2)	
Underweight	118	(3.4)	79	(66.9)	39	(33.1)		174	(4.0)	120	(69.0)	54	(31.0)	
**Number of chronic diseases**							<.0001							<.0001
0	1,961	(56.6)	802	(40.9)	1,159	(59.1)		2,449	(55.7)	1,307	(53.4)	1,142	(46.6)	
1	1,024	(29.6)	546	(53.3)	478	(46.7)		1,323	(30.1)	858	(64.9)	465	(35.1)	
≥2	478	(13.8)	297	(62.1)	181	(37.9)		621	(14.1)	474	(76.3)	147	(23.7)	

Table 3 presents the GEE model results of the association between changes in SA status and the risk of depressive symptoms. Overall, men and women had similar odds of experiencing depressive symptoms in the NSA→NSA group. Those in the NSA→NSA group, had the highest odds of depression in men (OR, 1.16; 95 % CI, 1.13–1.18) and in women (OR, 1.15; 95 % CI, 1.13–1.18). The odds of depression in the SA→NSA group were observed in men (OR, 1.11; 95 % CI, 1.08 − 1.13) and in women (OR, 1.11; 95 % CI, 1.09–1.14). Those in the NSA→SA group had the lowest risk of depression in men (OR, 1.03; 95 % CI, 1.00–1.05) and in women (OR, 1.06; 95 % CI, 1.03–1.08), despite this association not being statistically significant in men.

**Table 3:** Association between change in successful aging status and depressive symptoms

Variables	Depressive symptoms (CES-D10 ≥ 3)							
	**Men**				**Women**			
	Adjusted OR	95 % CI			Adjusted OR	95 % CI		
**Change in successful aging**								
NSA→ NSA	1.16	(1.13	-	1.18)	1.15	(1.13	-	1.18)
SA→NSA	1.11	(1.08	-	1.13)	1.11	(1.09	-	1.14)
NSA→SA	1.03	(1.00	-	1.05)	1.06	(1.03	-	1.08)
SA→SA	1.00		-		1.00		-	
**Age**								
45–54	1.00		-		1.00		-	
55–64	0.96	(0.94	-	0.99)	0.98	(0.96	-	1.00)
≥ 65	0.96	(0.93	-	0.99)	0.97	(0.94	-	1.00)
**Education Level**								
Lower than middle school	1.10	(1.06	-	1.14)	1.11	(1.06	-	1.16)
Middle school graduate	1.05	(1.01	-	1.09)	1.05	(1.00	-	1.10)
High School graduate	1.04	(1.01	-	1.08)	1.03	(0.98	-	1.08)
University graduate	1.00		-		1.00		-	
**Income level**								
Low	1.04	(1.02	-	1.07)	1.06	(1.04	-	1.09)
Middle Low	1.04	(1.02	-	1.07)	1.03	(1.01	-	1.06)
Middle High	1.01	(0.99	-	1.04)	1.01	(0.99	-	1.03)
High	1.00		-		1.00		-	
**Marital status**								
Married	1.00		-		1.00		-	
Unmarried	1.17	(1.13	-	1.20)	1.07	(1.05	-	1.10)
**Economic activity**								
Active	1.00		-		1.00		-	
Inactive	1.10	(1.07	-	1.12)	1.06	(1.04	-	1.08)
**Region**								
Urban	0.98	(0.96	-	1.00)	0.99	(0.97	-	1.01)
Rural	1.00		-		1.00		-	
**Physical activity**								
Yes	1.00		-		1.00		-	
No	1.05	(1.03	-	1.07)	1.05	(1.03	-	1.07)
**Drinking**								
Current	0.98	(0.95	-	1.00)	0.97	(0.94	-	0.99)
Past	1.04	(1.01	-	1.08)	1.01	(0.98	-	1.04)
Never	1.00		-		1.00		-	
**Smoking**								
Current	1.00	(0.97	-	1.02)	1.08	(1.03	-	1.14)
Past	0.98	(0.96	-	1.01)	1.00	(0.94	-	1.06)
Never	1.00		-		1.00		-	
**BMI**^**a**^								
Overweight	0.96	(0.94	-	0.98)	0.99	(0.97	-	1.01)
Normal	1.00		-		1.00		-	
Underweight	1.09	(1.05	-	1.14)	1.02	(0.99	-	1.06)
**Number of chronic diseases**								
0	1.00		-		1.00		-	
1	1.02	(1.00	-	1.05)	1.03	(1.01	-	1.05)
≥ 2	1.05	(1.02	-	1.08)	1.08	(1.05	-	1.10)

Note: Adjusted for other covariates

^a^*BMI* Body Mass Index

The independent subgroup analysis results of the variables associated with the effect of the changes in SA status on depression are shown in Table 4. Men who were ≥ 65 years of age and in the NSA→NSA group (OR, 1.20; 95 % CI, 1.17–1.24) had increased odds of depressive symptoms. Those who were past drinkers (OR, 1.19; 95 % CI, 1.13–1.26), past smokers (OR, 1.20; 95 % CI, 1.15–1.24), underweight (OR, 1.37; 95 % CI, 1.17–1.59), and in the NSA→NSA group had higher probabilities of having depressive symptoms than those in other groups. Furthermore, men in the NSA→NSA group who had more than two comorbidities had the greatest risks of elevated depressive symptoms (OR, 1.18; 95 % CI, 1.12–1.24. Similarly, women in the NSA→NSA group and were ≥ 65 years of age (OR, 1.18; 95 % CI, 1.14–1.21), current drinkers (OR, 1.22; 95 % CI, 1.17–1.28), current smokers (OR, 1.21; 95 % CI, 1.01–1.44), and underweight (OR, 1.25; 95 % CI, 1.12–1.39), were more likely to have depressive symptoms than those in other groups. In addition, women in the NSA→NSA group with more than two comorbidities (OR, 1.19; 95 % CI, 1.13–1.26) had the highest risks of depressive symptoms.

**Table 4:** The results of subgroup analysis stratified by covariates

Variable	Depressive symptoms (CES-D10 ≥ 3)												
	**Change in successful aging**												
	**SA→SA**	**NSA→SA**				**SA→NSA**				**NSA→ NSA**			
	**Adjusted OR**	**Adjusted OR**	**95 % CI**			**Adjusted OR**	**95 % CI**			**Adjusted OR**	**95 % CI**		
**Men**													
** Age**													
45–54	1.00	1.03	(0.98	-	1.08)	1.13	(1.07	-	1.19)	1.15	(1.08	-	1.22)
55–64	1.00	1.07	(1.03	-	1.10)	1.12	(1.08	-	1.16)	1.14	(1.11	-	1.18)
≥ 65	1.00	1.06	(1.02	-	1.09)	1.14	(1.10	-	1.17)	1.20	(1.17	-	1.24)
**Drinking**													
Current	1.00	1.04	(1.01	-	1.07)	1.11	(1.08	-	1.14)	1.17	(1.14	-	1.20)
Past	1.00	1.04	(0.99	-	1.11)	1.16	(1.10	-	1.23)	1.19	(1.13	-	1.26)
Never	1.00	1.27	(1.06	-	1.53)	1.12	(1.03	-	1.21)	1.02	(0.94	-	1.10)
**Smoking**													
Current	1.00	1.04	(1.00	-	1.08)	1.13	(1.09	-	1.18)	1.18	(1.13	-	1.23)
Past	1.00	1.01	(0.98	-	1.05)	1.11	(1.07	-	1.15)	1.20	(1.15	-	1.24)
Never	1.00	1.04	(1.00	-	1.08)	1.10	(1.06	-	1.14)	1.13	(1.09	-	1.17)
**BMI**^**a**^													
Overweight	1.00	1.02	(0.98	-	1.07)	1.13	(1.08	-	1.19)	1.18	(1.12	-	1.24)
Normal	1.00	1.02	(1.00	-	1.05)	1.10	(1.08	-	1.13)	1.15	(1.13	-	1.18)
Underweight	1.00	1.34	(1.12	-	1.60)	1.24	(1.02	-	1.52)	1.37	(1.17	-	1.59)
**Number of chronic diseases**													
0	1.00	1.03	(1.00	-	1.06)	1.12	(1.08	-	1.15)	1.15	(1.11	-	1.19)
1	1.00	1.05	(1.01	-	1.09)	1.11	(1.07	-	1.16)	1.15	(1.11	-	1.20)
≥ 2	1.00	1.01	(0.95	-	1.07)	1.10	(1.05	-	1.16)	1.18	(1.12	-	1.24)
**Women**													
** Age**													
45–54	1.00	1.03	(0.98	-	1.08)	1.13	(1.07	-	1.19)	1.15	(1.08	-	1.22)
55–64	1.00	1.07	(1.03	-	1.10)	1.12	(1.08	-	1.16)	1.14	(1.11	-	1.18)
≥65	1.00	1.07	(1.03	-	1.11)	1.12	(1.09	-	1.16)	1.18	(1.14	-	1.21)
**Drinking**													
Current	1.00	1.05	(1.01	-	1.10)	1.15	(1.10	-	1.21)	1.22	(1.17	-	1.28)
Past	1.00	1.08	(0.99	-	1.19)	1.12	(1.03	-	1.22)	1.16	(1.05	-	1.28)
Never	1.00	1.06	(1.03	-	1.09)	1.10	(1.08	-	1.13)	1.14	(1.11	-	1.17)
**Smoking**													
Current	1.00	0.99	(0.77	-	1.28)	1.19	(0.99	-	1.42)	1.21	(1.01	-	1.44)
Past	1.00	1.09	(0.90	-	1.33)	1.13	(0.94	-	1.36)	1.08	(0.91	-	1.29)
Never	1.00	1.06	(1.03	-	1.08)	1.11	(1.09	-	1.14)	1.15	(1.13	-	1.18)
**BMI**^**a**^													
Overweight	1.00	1.06	(1.02	-	1.11)	1.10	(1.06	-	1.14)	1.15	(1.10	-	1.19)
Normal	1.00	1.05	(1.03	-	1.08)	1.11	(1.09	-	1.14)	1.16	(1.13	-	1.19)
Underweight	1.00	0.98	(0.86	-	1.13)	1.30	(1.15	-	1.48)	1.25	(1.12	-	1.39)
**Number of chronic diseases**													
0	1.00	1.06	(1.03	-	1.09)	1.12	(1.09	-	1.15)	1.15	(1.12	-	1.19)
1	1.00	1.06	(1.02	-	1.10)	1.11	(1.07	-	1.15)	1.16	(1.12	-	1.20)
≥ 2	1.00	1.06	(1.00	-	1.13)	1.12	(1.06	-	1.19)	1.19	(1.13	-	1.26)

Note:Adjusted for other covariates

^a^*BMI* Body Mass Index

Table 5 shows the longitudinal analysis results of the effect of each component of the two-year change in SA status on the risk of depressive symptoms. Men with onset of ADL limitations (OR, 1.27; 95 % CI, 1.22–1.32), no change in IADL limitations (OR, 1.14; 95 % CI, 1.11–1.18), continuous non-participation in social activities (OR, 1.12; 95 % CI, 1.09–1.15), no improvement in cognitive functioning (OR, 1.19; 95 % CI, 1.16–1.23), and consistent low physical functioning (OR, 1.13; 95 % CI, 1.11–1.16), had statistically significant increased risks of depressive symptoms. Similarly, women with onset of ADL limitations (OR, 1.17; 95 % CI, 1.09–1.20), no change in IADL limitations (OR, 1.16; 95 % CI, 1.12–1.20), continuous non-participation in social activities (OR, 1.07; 95 % CI, 1.04–1.09), no improvement in cognitive functioning (OR, 1.15; 95 % CI, 1.12–1.17), and continuous low physical functioning (OR, 1.13; 95 % CI, 1.11–1.15), had the greatest risks of depressive symptoms.

**Table 5:** Subgroup analysis of change in each component of successful aging with depressive symptoms

Variables	Depressive symptoms (CES-D10 ≥ 3)							
	**Men**				**Women**			
	Adjusted OR	**95 % CI**			Adjusted OR	**95 % CI**		
**Change in ADL limitations**								
Yes→Yes	1.22	(1.15	-	1.30)	1.14	(1.09	-	1.20)
No→Yes	1.27	(1.22	-	1.32)	1.17	(1.13	-	1.21)
Yes→No	1.12	(1.05	-	1.19)	1.03	(0.99	-	1.08)
No→No	1.00		-		1.00		-	
**Change in IADL limitations**								
Yes→Yes	1.14	(1.11	-	1.18)	1.16	(1.12	-	1.20)
No→Yes	1.11	(1.08	-	1.14)	1.14	(1.11	-	1.17)
Yes→No	1.01	(0.98	-	1.04)	0.99	(0.97	-	1.02)
No→No	1.00		-		1.00		-	
**Change in social activity particpation**								
No→No	1.12	(1.09	-	1.15)	1.07	(1.04	-	1.09)
Yes→No	1.09	(1.06	-	1.12)	1.06	(1.04	-	1.08)
No→Yes	1.06	(1.03	-	1.08)	1.04	(1.02	-	1.06)
Yes→Yes	1.00		-		1.00		-	
**Change in high cognitive functioning**								
No→No	1.19	(1.16	-	1.23)	1.15	(1.12	-	1.17)
Yes→No	1.14	(1.11	-	1.17)	1.12	(1.10	-	1.14)
No→Yes	1.07	(1.04	-	1.10)	1.06	(1.04	-	1.09)
Yes→Yes	1.00		-		1.00		-	
**Change in high physical functioning**								
No→No	1.13	(1.11	-	1.16)	1.13	(1.11	-	1.15)
Yes→No	1.10	(1.08	-	1.12)	1.11	(1.08	-	1.13)
No→Yes	1.04	(1.02	-	1.06)	1.05	(1.02	-	1.07)
Yes→Yes	1.00		**-**		1.00		**-**	

Note:Adjusted for other covariates

## Discussion

Our study presented a new viewpoint to further comprehend the multifaceted nature of successful aging by longitudinally examining its association with depressive symptoms. Our findings reported that a change of SA to NSA or consistent NSA over a span of two years predicted a higher risk of depressive symptoms in middle aged and older adults after two years. Notably, the prevalence of depressive symptoms was higher in women than in men, particularly in the NSA→NSA group (74 % in women vs. 62.2 % in men). Although evidence has been inconsistent, previous research has suggested that, particularly in women, an inverse relationship exists between SA and depression [24]. The explanation offered was that women were more likely to report depressive symptoms and less likely to successfully age [25], thereby playing a potential role in gender disparities. Despite this, our study did not find any marked sex differences in reported ORs of association between SA and depressive symptoms, suggesting that 2-year SA changes had similar influences on depressive symptoms in both Korean older men and women.

Cross-study comparisons are difficult due to variations in successful aging criteria and lack of longitudinal studies; however, previous evidence suggests an inverse cross-sectional relationship between SA and depression. A descriptive study by Shin et al. investigated the correlation between SA, measured using a Korean elderly successful aging scale, and depression in elderly women, noting that the two factors had a significant inverse correlation [26]. A study conducted in Brazil used active aging, a concept similar to successful aging, having a common feature of social activity participation, stated that older adults who actively aged had a lower prevalence of depression after controlling for multiple confounders [27].While the exact mechanism behind the relationship between SA and depression remains unknown, we hypothesized that compared to those met criteria for SA, those who could not meet criteria for SA feel discouraged and hopeless due to the accumulation of multiple burdens (such as cognitive decline, disability, and limited social circle and activity), thereby causing feelings of depression [28].

Among those who consistently did not meet the Rowe and Kahn criteria for SA, those in the oldest age group had the highest risk of depressive symptoms, in both men and women. The highest risk of depression in the ≥ 65 years age group could be attributed to the aging process, inability to age successfully, and worsening health, thereby negatively affecting the psychological state of mind [29], leading to depressive symptoms. NSA in men and women, who were current drinkers and current smokers, exhibited high risks of depressive symptoms. The influence of drinking and smoking on depression in the elderly has been established in previous studies [30, 31]. Furthermore, the relationship between alcohol consumption, successful aging, and depression has been previously deemed important [28], with those not meeting SA criteria turning to alcohol consumption to cope with dissatisfaction and depression. One study linked NSA with low BMI [32], while another showed that older adults who were underweight were more likely to experience depressive symptoms than their normal or overweight counterparts [33, 34].

In our gender-stratified subgroup analysis, multimorbidity showed similar results in both men and women. The presence of more chronic diseases was positively associated with depression in non-successful aging in men and women. In contrast, a meta-analysis study by Kim et al. reported that comorbidities did not greatly affect SA [35]. The authors attempted to explain this by stating that most of the Korean aging population are more likely to have one or more chronic diseases, and that individuals are more reliant on their cognitive and physical functionality during aging [35]. Another study also concluded that the presence of chronic illness is not to be equated with “non successful aging,” as older adults’ perception of successful aging changes [36], indicative of their health-seeking behaviors [37]. This notion is also supported by the WHO, which stated that health-related policy goals should shift from the former approach revolving around the prevention of disease, to a more positive concept of successful aging [38]. Despite this, our study’s results revealed the essential influence of increased multimorbidity on depressive symptoms in older adults, and subsequently considered successful aging analyses.

Despite the variations in the conceptualization of successful aging, an established feature of SA is that it is a complex, multidimensional phenomenon that is not only influenced by individual factors such as high physical and mental functioning but also involves an interplay of multiple variables at once [39]. Nevertheless, we attempted to discern and compare the longitudinal influence of the components of SA on depressive symptoms in both genders. Of all the dimensions of SA, both men and women showed great risks of depressive symptoms during the onset of ADL limitations in a span of two years. A prior finding revealed that the relationship between ADL limitation and SA is mediated by depression, and SA interventions are required to improve ADL and reduce depression [40]. Furthermore, our results mirror those of previous studies that reviewed the literature on the most common contributing factors of SA. The influence of disability, cognitive functioning, and physical functioning—three recurring variables that appear in nearly every definition of SA—on depressive symptoms in men and women highlights the importance of including these variables in successful aging models [6, 18].

Moreover, our study’s main strength lies in the fact that it is one of the few studies that investigated the effect of prospective SA changes on depression, using a longitudinal form of the Rowe and Kahn model. Despite the existing literature on SA and its mental health consequences, to the best of our knowledge, our study is the first in Korea and one of the few worldwide to have explored the effect of longitudinal SA changes on depressive symptoms. Second, we used nationally representative data from the KLoSA, one of the largest panel surveys on the elderly population in South Korea.

Despite these strengths, our study is not without limitations. First, although we used a longitudinal model to detect changes over time, we could not confirm the directionality and causality of the relationship between SA and depressive symptoms. For example, Kim et al. explored the association between depressive symptoms and SA, among other factors, and the results revealed that depression was the most influential factor on SA [17]. Second, although we used the widely popular Rowe and Kahn model, the use of several domains to define SA is rather limiting. Prior studies have argued that the definition of SA is too strict and may set unrealistic expectations for aging in older populations [41].

## Conclusions

In conclusion, successful aging is determined through multiple factors, and its influence on depression involves complex interactions that accumulate over time. Our study’s findings showed that older adults who did not meet the criteria for SA over a period of two years had the highest risk of depressive symptoms, followed by those classified as initially meeting the SA criteria but did not meet the criteria two years later. Based on our findings, it is of utmost importance to develop interventions and promotion strategies advocating against depression and maintaining a healthier and more successful aging process, tailored to the South Korean aging demographic.

## Data Availability

The data used in this study was the KLoSA conducted by the Korea Employment Information Service (KEIS) and is publicly available at https://survey.keis.or.kr/eng/klosa/databoard/List.jsp.

## Abbreviations

GEE: Generalized Estimating Equation
OR: Odds Ratio
SA: Successful Aging
ADL: Activities of Daily Living
IADL: Instrumental Activities of Daily Living
KLoSA: Korean Longitudinal Study of Aging
K-MMSE: Korean Mini-Mental State Examination
BMI: Body mass index
CES-D10: 10-item Center for the Epidemiological Studies of Depression Short Form

## References

[1] Ouwehand C, de Ridder DT, Bensing JM (2007). A review of successful aging models: Proposing proactive coping as an important additional strategy. Clin Psychol Rev.

[2] McLaughlin SJ, Jette AM, Connell CM (2012). An examination of healthy aging across a conceptual continuum: prevalence estimates, demographic patterns, and validity. J Gerontol A Bio Sci Med Sci.

[3] Rowe JW, Kahn RLJS (1987). Human aging: Usual and successful. Science.

[4] Von Faber M, Bootsma–van der Wiel A, Van Exel E, Gussekloo J, Lagaay AM, van Dongen E (2001). Successful aging in the oldest old: Who can be characterized as successfully aged?. Arch Intern Med.

[5] Domènech-Abella J, Perales J, Lara E, Moneta MV, Izquierdo A, Rico-Uribe LA (2018). Sociodemographic factors associated with changes in successful aging in Spain: A Follow-Up Study. J Aging Health.

[6] Kok AAL, Aartsen MJ, Deeg DJH, Huisman M (2015). Capturing the diversity of successful aging: An operational definition based on 16-year trajectories of functioning. Gerontologist.

[7] Stowe JD, Cooney TM (2014). Examining Rowe and Kahn’s concept of successful aging: Importance of taking a life course perspective. Gerontologist.

[8] Rowe JW, Kahn RL (2015). Successful Aging 2.0: Conceptual expansions for the 21st century. J Genrontol B.

[9] Dahany MM, Dramé M, Mahmoudi R, Novella JL, Ciocan D, Kanagaratnam L (2014). Factors associated with successful aging in persons aged 65 to 75 years. Eur Geriatr Med.

[10] Bosnes I, Nordahl HM, Stordal E, Bosnes O, Myklebust T, Almkvist O (2019). Lifestyle predictors of successful aging: A 20-year prospective HUNT study. PloS one.

[11] Kim H-J, Min J-Y, Min K-B (2019). Successful aging and mortality risk: The Korean longitudinal study of aging (2006–2014). J Am Med Dir Assoc.

[12] Kim M (2017). The effect of successful aging on the life satisfaction of Korean older adults. Innov Aging.

[13] Nakagawa T, Cho J, Yeung D (2020). Successful Aging in East Asia: Comparison among China, Korea, and Japan. J Gerontol B.

[14] Dilip J, Depp C. Strategies for Successful Aging: A Research Update. Current Psychiatry Reports. 2014;16(10). 10.1007/s11920-014-0476-6 PMC4207365 25135776

[15] Jin Y, Cho J, Lee I, Park S, Kim D, Kong J (2020). Involuntary weight loss and late-life depression in Korean older adults. Iran J Public Health.

[16] Park JH, Kim Ki WK. A review of the epidemiology of depression in Korea. Journal of the Korean Medical Association.2011;54.

[17] Kim YS, Cho JH (2020). The influence of social network and depression on successful aging in the elderly. Medico Legal Update.

[18] Depp CA, Jeste DV (2006). Definitions and predictors of successful aging: a comprehensive review of larger quantitative studies. The American Journal of Geriatric Psychiatry.

[19] Radloff LS. The CES-D scale: A self-report depression scale for research in the general population. 1977;1:385–401.

[20] Andresen EM, Malmgren JA, Carter WB, Patrick DL (1994). Screening for depression in well older adults: Evaluation of a short form of the CES-D (Center for Epidemiologic Studies Depression Scale). Am J Prev Med.

[21] Irwin M, Artin KH, Oxman MN (1999). Screening for depression in the older adult: Criterion validity of the 10-item Center for Epidemiological Studies Depression Scale (CES-D). Arch Intern Med.

[22] Kokko K, Feldt T (2018). Longitudinal profiles of mental well-being as correlates of successful aging in middle age. Int J Behav Dev.

[23] Jang HY (2020). Factors associated with successful aging among community-dwelling older adults based on ecological system model. IJERPH.

[24] Golja K, Daugherty AM, Kavcic V (2020). Cognitive reserve and depression predict subjective reports of successful aging. Arch Genrontol Geriatr.

[25] Cheng S-T (2014). Defining successful aging: The need to distinguish pathways from outcomes. Int Psychogeriatr.

[26] Shin SH, Jang KS, Choi O (2019). Study of the Successful Aging of the Elderly Women in Rural Area. J Health Info Stat.

[27] Galli R, Moriguchi EH, Bruscato NM, Horta RL, Pattussi MP (2016). Active aging is associated with low prevalence of depressive symptoms among Brazilian older adults. Rev Bras Epidemiol.

[28] Bernhold QS, Gasiorek J (2020). Older adults’ perceptions of their own and their romantic partners’ age-related communication and their associations with aging well, depressive symptoms, and alcohol use disorder symptoms. Journal of social personal relationships.

[29] Kok RM, Reynolds CF (2017). Management of Depression in Older Adults: A Review. JAMA.

[30] Li J, Wang H, Li M, Shen Q, Li X, Zhang Y (2020). Effect of alcohol use disorders and alcohol intake on the risk of subsequent depressive symptoms: a systematic review and meta-analysis of cohort studies. Addiction.

[31] An R, Xiang X (2015). Smoking, heavy drinking, and depression among US middle-aged and older adults. Preventive medicine.

[32] Luo H, Ren X, Li J, Wu K, Wang Y, Chen Q (2020). Association between obesity status and successful aging among older people in China: Evidence from CHARLS. BMC Public Health.

[33] Kim J, Noh J-W, Park J, Kwon YD (2014). Body Mass Index and Depressive Symptoms in Older Adults: A Cross-Lagged Panel Analysis. PLOS ONE.

[34] Estrella-Castillo E, Gómez-de-Regil DF. L. Comparison of body mass index range criteria and their association with cognition, functioning and depression: A cross-sectional study in Mexican older adults. BMC Geriatr. 2019;19:339. 10.1186/s12877-019-1363-0 PMC6889317 31795994

[35] Kim S-H, Park S, Park K-S (2017). Correlates of Successful Aging in South Korean Older Adults: A Meta-Analytic Review. Phys Rev.

[36] Collis D, Waterfield J (2015). The understanding of pain by older adults who consider themselves to have aged successfully. Musculoskeletal Care.

[37] Bowling A (2006). Lay perceptions of successful ageing: findings from a national survey of middle aged and older adults in Britain. Eur J Ageing.

[38] ‎‎ World Health Organization. A glossary of terms for community health care and services for older persons. Kobe: WHO Centre for Health Development; 2004.

[39] Kozar-Westman M, Troutman-Jordan M, Nies MA (2013). Successful aging among assisted living community older adults. J Nurs Scholarsh.

[40] Choi J-M, Yang J-B (2017). A structural equation modeling analysis of the relationships between activities of daily living, depression, and successful aging among the Korean elderly with disabilities. International Information Institute (Tokyo) Information.

[41] Martinson M, Berridge C (2014). Successful aging and its discontents: A systematic review of the social gerontology literature. Gerontologist.

